# Modulation of the subthalamic nucleus activity by serotonergic agents and fluoxetine administration

**DOI:** 10.1007/s00213-013-3333-0

**Published:** 2013-11-24

**Authors:** A. Aristieta, T. Morera-Herreras, J. A. Ruiz-Ortega, C. Miguelez, I. Vidaurrazaga, A. Arrue, M. Zumarraga, L. Ugedo

**Affiliations:** 1 Department of Pharmacology, Faculty of Medicine and Dentistry, University of the Basque Country UPV/EHU, 48940 Leioa, Spain; 2 Department of Pharmacology, Faculty of Pharmacy, University of the Basque Country UPV/EHU, 01006 Vitoria-Gasteiz, Spain; 3 Red de Salud Mental de Bizkaia. Departamento de Investigación Neuroquímica, Hospital de Zamudio, Arteaga Auzoa, 45, 48170 Zamudio, Spain

**Keywords:** pCPA, Subthalamic nucleus, Serotonin, Fluoxetine, Single-unit extracellular recording, Extrapyramidal effects

## Abstract

**Rationale:**

Within the basal ganglia, the subthalamic nucleus (STN) is the only glutamatergic structure and occupies a central position in the indirect pathway. In rat, the STN receives serotonergic input from the dorsal raphe nucleus and expresses serotonergic receptors.

**Objective:**

This study examined the consequences of serotonergic neurotransmission modulation on STN neuron activity.

**Methods:**

In vivo single-unit extracellular recordings, HPLC determination, and rotarod and bar test were performed in control, 4-chloro-DL-phenylalanine methyl ester hydrochloride- (pCPA, a serotonin synthesis inhibitor) and chronically fluoxetine-treated rats.

**Results:**

The pCPA treatment and the administration of serotonin (5-HT) receptor antagonists increased number of bursting neurons in the STN. The systemic administration of the 5-HT_1A_ agonist, 8-OH-DPAT, decreased the firing rate and increased the coefficient of variation of STN neurons in pCPA-treated rats but not in control animals. Additionally, microinjection of 8-OH-DPAT into the STN reduced the firing rate of STN neurons, while microinjection of the 5-HT_2C_ agonist, Ro 60-0175, increased the firing rate in both control and fluoxetine-treated animals. Finally, the fluoxetine challenge increased the firing rate of STN neurons in fluoxetine-treated rats and induced catalepsy.

**Conclusions:**

Our results indicate that the depletion and the blockage of 5-HT modify STN neuron firing pattern. STN neuron activity is under the control of 5-HT_1A_ and 5-HT_2C_ receptors located both inside and outside the STN. Finally, fluoxetine increases STN neuron activity in chronically fluoxetine-treated rats, which may explain the role of this nucleus in fluoxetine-induced extrapyramidal side effects.

**Electronic supplementary material:**

The online version of this article (doi:10.1007/s00213-013-3333-0) contains supplementary material, which is available to authorized users.

## Introduction

The serotonergic system is widely distributed through the brain and modulates multiple central nervous system functions such as motor activity (Benarroch 2009). Several clinical reports have highlighted that the chronic treatment with selective serotonin (5-HT) reuptake inhibitors (SSRIs), such as fluoxetine, can induce extrapyramidal side effects (e.g., tardive dyskinesia or parkinsonism), akathisia and/or dystonia (Bilen et al. 2008; Leo 1996). Studies performed in neonatal rats have also shown that fluoxetine treatment reduces locomotor activity (Lee 2012). These side effects may occur because chronic 5-HT transporter blockage alters the functionality of the basal ganglia (BG) (Morelli et al. 2011). In fact, the BG receive serotonergic innervation arising mainly from the dorsal raphe nucleus (Di Matteo et al. 2008; Liu et al. 2007).

The BG form a highly organized network that connects the thalamus with the cortex and plays an important role in the regulation of motor behavior. In this circuit, the subthalamic nucleus (STN) is the sole glutamatergic nucleus and it exerts the principal glutamate-mediate excitatory effect upon the output structures of the BG. Dysregulation of STN neuron activity has been related to motor disturbances (Crossman 2000; Obeso et al. 2000). As other BG nuclei, the STN receives serotonergic innervation and contains 5-HT and its metabolite, 5-hydroxy-indolacetic acid (5-HIAA) (Lavoie and Parent 1990; Palkovits et al. 1974; Saavedra 1977). Expression of 5-HT_1A_, 5-HT_2C_, and 5-HT_4_ mRNA and binding sites have also been shown in STN cells (Eberle-Wang et al. 1997; Grossman et al. 1993; Maroteaux et al. 1992; Pazos et al. 1985; Pazos and Palacios 1985; Pompeiano et al. 1992; Waeber et al. 1996). Moreover, 5-HT_1B/D_ receptors are present in the terminals of neurons that project to the STN from the motor cortex and the external segment of the globus pallidus (Hannon and Hoyer 2008; Hoyer et al. 2002; Sari et al. 1999).

Using in vitro recording techniques, it has been shown that 5-HT increases the spontaneous firing rate of STN neurons via 5-HT_2C_ and 5-HT_4_ receptors (Flores et al. 1995; Shen et al. 2007; Stanford et al. 2005; Xiang et al. 2005) and produces the opposite effect through 5-HT_1A_ receptors (Shen et al. 2007; Stanford et al. 2005). On the other hand, non-selective 5-HT_1_ or 5-HT_2_ receptor agonists microinjected into the STN cause hyperlocomotion in rats (Belforte and Pazo 2004; Martinez-Price and Geyer 2002), whereas a 5-HT_2C_ agonist microinjection induces orofacial dyskinesia (De Deurwaerdere and Chesselet 2000; Eberle-Wang et al. 1996). All these findings reveal an interaction between the serotonergic system and the STN. However, the effect of 5-HT neurotransmission modulation or 5-HT receptor activation on STN neuron activity in vivo is still unknown.

In the present study, we aimed to determinate the in vivo serotonergic modulation of the electrical activity of the STN by applying serotonergic agonists and antagonists to control, 4-chloro-DL-phenylalanine methyl ester hydrochloride (pCPA, a 5-HT synthesis inhibitor) and fluoxetine-treated rats and to evaluate the possible impact of fluoxetine treatment on motor behavior and STN activity. To accomplish this, both electrophysiological and behavioral approaches were used. Our results show that modulation of endogenous 5-HT modifies the firing pattern and 5-HT receptor-mediated response of STN.

## Material and methods

### Animals

Male Sprague–Dawley rats (225–325 g) were housed under a 12:12 h light:dark cycle with food and water provided ad libitum. Every effort was made to minimize animal suffering and to use the minimum number of animals possible. Experimental protocols were approved by the Local Ethical Committee for Animal Research at the University of the Basque Country. All of the experiments were performed in compliance with the European Community Council Directive on “The Protection of Animals Used for Experimental and Other Scientific Purposes” (86/609/EEC) and with Spanish Law (RD 1201/2005) for the care and use of laboratory animals. Four experimental groups of animals were used in this study: pCPA-treated rats (5-HT synthesis inhibitor, 300 mg/kg, i.p., 3 days; *n* = 43) (Ugedo et al. 1989), fluoxetine-treated rats (SSRI, 10 mg/kg, i.p., 14 days; *n* = 24) (Guirado et al. 2009) and their respective vehicle-treated control rats (*n* = 118).

### Behavioral testing

#### Rotarod

The rotarod test was performed to detect potential impairment of motor performance and coordination in the rats (Dekundy et al. 2006). Animals were pre-trained for four consecutive days on an automated four-lane rotarod unit (Panlab, Spain) that accelerated smoothly from 4 to 40 rpm over a period of 300 s. Every training day, three trials were performed with a rest of 30 min between each trial to avoid fatigue. Once animals had reached a stable baseline performance, chronic treatment with fluoxetine or vehicle was started and testing sessions were carried out on days 1, 5, 9, and 14 after the beginning of the treatments. On each testing day, animals were evaluated three times in the accelerating protocol over 300 s at three different time points, just before, 30 and 60 min after drug administration. The time to fall off the rod at each testing time was recorded.

#### Bar test

The bar test was carried out to measure catalepsy along the chronic treatment with fluoxetine or vehicle on days 1, 5, 9, and 14. The latency to initiate the movement was used as measure of catalepsy (Dekundy et al. 2006). On each testing day, the animals were evaluated on two consecutive trials at three different time points just before, 30 and 60 min after drug administration, preceding the performance on the rotarod. On the testing trials, the animal was gently placed with both forepaws on a 9-cm-high standard wooden bar. The end point of catalepsy was considered when both forepaws were removed from the bar. The maximal testing duration was set at 120 s.

### Electrophysiological procedures

The electrophysiological recording of STN neurons was performed as described by Morera-Herreras et al. (2010). Rats were anesthetized with urethane (1.2 g/kg, i.p.), and the right jugular vein was cannulated for additional drug injections. The rat was placed in a stereotaxic frame with its head secured in a horizontal orientation. The recording electrode was lowered into the right STN (relative to bregma and dura: AP −3.6/−3.8 mm, ML −2.2/−2.7 mm, and DV −7/−8.5 mm (Paxinos and Watson 1997). The extracellular signal from the electrode was amplified with a high-input impedance amplifier and was monitored on an oscilloscope and on an audio monitor. All recorded STN neurons exhibited a biphasic waveform and a pulse width of 1.0 to 1.5 ms (Hollerman and Grace 1992). Neuronal spikes were digitized using computer software (CED micro 1401 interface and Spike2 software, Cambridge Electronic Design). The basal firing rate was recorded for 5–10 min.

At the end of each electrophysiological experiment, a Pontamine Sky Blue mark was deposited. The recording site was located in brain slices by neutral red staining (Miguelez et al. 2011), both when the administration was performed intravenously or locally. Only cells recorded within the STN were included in this study.

The firing parameters of STN neurons were analyzed off-line using Spike2 software. The following parameters were calculated: firing rate and coefficient of variation. According to the method described by Kaneoke and Vitek (1996), three different firing patterns could be analyzed (using the NeuronFit program from NorayBio Informatics): (1) a tonic firing pattern, (2) a random firing pattern, and (3) a bursting firing pattern. The analyzed spike-trains lasted more than 120 s and contained at least 300 spikes.

### Pressure microinjection into the subthalamic nucleus

A calibrated pipette glued adjacent to a recording micropipette (Ruiz-Ortega and Ugedo 1997) was filled either with 5 μM of the 5-HT_1A_ receptor agonist 8-OH-DPAT, 20 mM of the 5-HT_2C_ receptor agonist Ro 60-0175, 10 μM of the 5-HT_1A_ receptor antagonist WAY 100635, or 100 μM of the 5-HT_2C_ receptor antagonist SB 242084. Drugs were dissolved in Dulbecco's buffered saline solution containing (in mM): NaCl 136.9, KCl 2.7, NaH_2_PO_4_ 8.1, KH_2_PO_4_ 1.5, MgCl_2_ 0.5, and CaCl_2_ 0.9 (pH = 7.40), with the exception of SB 242084 that was dissolved in Dulbecco's buffered saline solution containing 8 % hydroxypropyl-β-cyclodextrin. Drug injection was performed by applying pressure pulses (50–150 ms) with a Picospritzer™ II (General Valve Corporation, Fairfield, NJ, USA). The injected volume was measured by monitoring the meniscus movement in a calibrated pipette. Every pipette was calibrated so that each pulse corresponded to the injection of 2 nl of solution.

### High-performance liquid chromatography determination

For determination of 5-HT, 5-HIAA, and dopamine (DA), rats were deeply anesthetized with urethane (1.2 g/kg, i.p.) and decapitated. The brain was immediately extracted and the STN was specifically dissected using a microtome with a vibrating blade (Micron HM 650 V). The tissue was homogenized in 0.1 M perchloric acid at a concentration of 50 μg of tissue per microliter and centrifuged (30 min, 12,500×*g*), and the supernatant was spin-filtered. Finally, one aliquot corresponding to 1 mg of tissue was assessed by high-performance liquid chromatography with amperometric detection. The detector was operated at 0.6 V potential between the working electrode and the Ag/AgCl reference electrode at a sensitivity of 2 nA full-scale deflection. The mobile phase consisted of 2 l of 0.02 M citric acid and 1 L of 0.02 M disodium phosphate (pH = 3.1), containing 0.05 μM EDTA, 3.64 mM heptanosulphonic acid, and 15 % methanol (*v*/*v*). The column was a 250 × 4.6 mm Symmetry C18, 5 μM particle size column (Waters Corporation). All separations were achieved isocratically at room temperature. The flow rate was 1 ml/min.

A calibration curve was performed daily within a concentration range of 0.1 to 0.9 ng of each standard per injection. The concentrations of 5-HT, 5-HIAA, and DA in the tissue samples were calculated by extrapolation of the samples’ peak heights to the calibration curve.

### 5-HT transporter (SERT) immunohistochemistry

For determination of SERT, rats were deeply anesthetized with urethane (1.2 g/kg, i.p.) and decapitated. Sections through the prefrontal cortex, the hippocampus, and the STN containing both hemispheres of all animals were processed at the same time using precisely the same experimental conditions to minimize methodological variability. For this procedure, the brains were cut in 40-μm slices by means of a freezing microtome (Model HM 430, Microm®, Spain), sections were first incubated for 30 min in 30 % methanol in 0.1 M PB and 30 % hydrogen peroxide (H_2_O_2_) and then rinsed with 0.1 M PB for 5 min and placed in 1 % sodium borohydride (Sigma-Aldrich Química, Spain) for 30 min. Afterwards, the sections were profusely washed with PB before rinsing in 0.1 M Trizma base saline (TS; Sigma-Aldrich Química, Spain) for 10 min. Brain sections were then incubated in 0.5 % bovine serum albumin (Sigma-Aldrich Química, Spain) in 0.1 m TS and 0.25 % Triton X-100 (Sigma-Aldrich Química, Spain) for 30 min and right after in primary antibody (rabbit anti-SERT, 1: 2500, Immunostar, Hudson, WI, USA) for 48 h at room temperature. The sections were rinsed in 0.1 m TS for 30 min and incubated in 1:400 dilutions of biotinylated donkey anti-rabbit IgG (Jackson Immunoresearch, Stratech Scientific, Soham, UK) for 2 h at room temperature. Sections were rinsed with 0.1 M TS for 30 min followed by incubation for 30 min in avidin-biotin peroxidase complex (Vector Laboratories, Peterborough, UK). The peroxidase reaction product was visualized by incubation in a solution containing 0.022 % 3,3′diaminobenzidine (Sigma-Aldrich Química, Spain) and 0.003 % H_2_O_2_ for 10–11 min, as described previously (Rodriguez et al. 2008; Rodriguez et al. 2009). The reaction was stopped by rinsing the sections in 0.1 M TS for 6 min followed by 0.1 M PB for 15 min. Brain sections were mounted onto gelatin-coated slides, dehydrated in ascending concentration of ethanol (50, 70, 80, 90, 95, and 100 %), cleared with xylene and coverslipped.

### Optical density measurement

The expression and density of SERT labeling on vehicle-control and pCPA-treated animals were analyzed by measuring its optical density (OD) as previously described (Cordero et al. 2005). Images were taken rostral-caudally and mean optical density was visualized using image analysis NIH-produced software, ImageJ (http://rsb.info.nih.gov/nih.image/default.html). The OD was calculated from a relative scale of intensity. The calibration density was kept constant for measuring all sections to avoid experimental variances. Non-specific OD in sections was measured from the corpus callosum, these density values were used as background. SERT density of each area was measured independently and a single measurement was obtained from every area in each hemisphere. Measurement of mean density was taken and averaged, after background subtraction, from each area in both the left and the right hemisphere of each slice. The results are shown as SERT optical density (OD/pixel).

### Drugs

The following drugs were used in this study: 4-chloro-DL-phenylalanine methyl ester hydrochloride (pCPA), (±)-8-Hydroxy-2-(dipropylamino) tetralin hydrobromide (8-OH-DPAT), WAY 100635 and Ro 60-0175 from Sigma-Aldrich Química (Spain); methiothepin maleate, SB 242084 from Tocris Bioscience (Spain); and fluoxetine from Biotrend (Germany). For systemic administration, 8-OH-DPAT, WAY 100635, methiothepin maleate, and pCPA were prepared in physiological saline (NaCl 0.9 %); SB 242084 was prepared in physiological saline containing 8 % hydroxypropyl-β-cyclodextrin; Ro 60-0175, fluoxetine and urethane were prepared in distilled water. For local administration, 8-OH-DPAT, Ro 60-0175, and WAY 100635 were dissolved in Dulbecco’s buffered saline solution. SB 242084 was dissolved in Dulbecco’s buffered saline solution containing 8 % hydroxypropyl-β-cyclodextrin. With the exception of urethane, drugs were freshly prepared prior to immediate use.

### Statistical analysis of data

Experimental data were analyzed using the computer program GraphPad Prism (v. 5.01, GraphPad Software, Inc). The electrophysiological and neurochemical data were analyzed by unpaired Student’s *t* test, one-way analysis of variance (ANOVA) or Fisher’s exact test for the comparison of the number of cells with burst firing activity. The effect of the different drugs on the electrophysiological parameters was analyzed by one-way ANOVA (when some data were missing) or repeated measures (RM) one-way and RM two-way ANOVA. For behavioral experiments, RM two-way ANOVA were used. All significant ANOVAs were followed by Bonferroni’s post hoc test. The level of statistical significance was set at *p* < 0.05. Data are presented as the group mean ± standard error of the mean (SEM).

## Results

### Effect of the pCPA treatment, the fluoxetine treatment, and the non-selective serotonergic receptor antagonist administration on STN

Three hundred and ninety-one neurons were recorded in the STN 24 h after the last vehicle, pCPA (300 mg/kg, i.p. for 3 days) or fluoxetine (10 mg/kg, i.p. for 14 days) injection: 176 neurons from 41 vehicle-treated control rats, 85 neurons from 16 pCPA-treated rats, and 130 neurons from 17 fluoxetine-treated rats. All cells were located within the STN and showed a biphasic waveform with a duration of 1.0–1.5 ms.

**Fig. 1:**
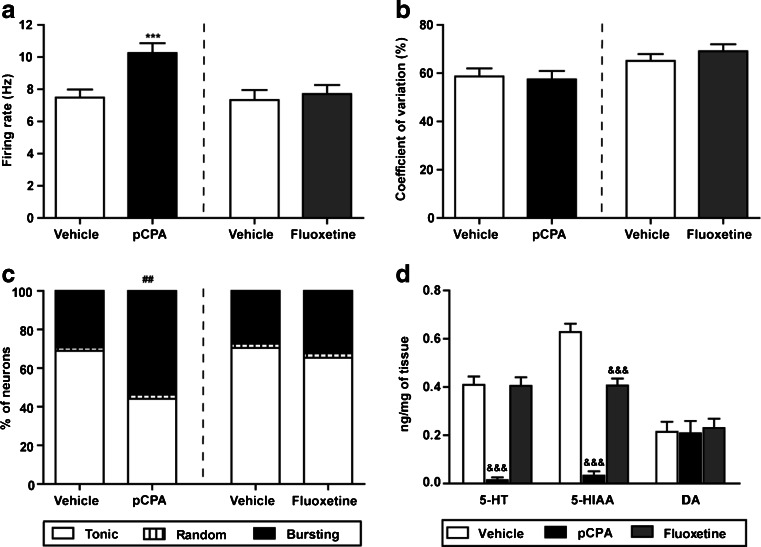
STN neuron electrophysiological parameters and 5-HT, 5-HIAA, and DA levels in the STN. Firing rate, coefficient of variation, and firing pattern (**a**, **b**, and **c**, respectively) of neurons recorded 24 h after the last vehicle, pCPA (300 mg/kg/day, i.p. 3 days) or fluoxetine (10 mg/kg/day, i.p. 14 days) injection; 86 neurons from 25 vehicle-control rats, 85 neurons from 16 pCPA-treated rats, 90 neurons from 16 vehicle-treated rats, and 130 neurons from 17 fluoxetine-treated rats. **d** Effect of pCPA (300 mg/kg/day, i.p., 3 days) and fluoxetine (10 mg/kg/day, i.p., 14 days) treatments on 5-HT, 5-HIAA, and DA levels in the STN, in control (*n* = 6), pCPA-treated (*n* = 5), and fluoxetine-treated (*n* = 6) animals. Each value represents the mean ± SEM. ***p < 0.001 vs. its control group (unpaired Student’s *t* test). ^##^ *p* < 0.01 vs. its control group (Fisher’s exact test). &&&*p* < 0.001 vs. control group (Bonferroni’s post hoc test)

As shown in Fig. 1a, the mean basal firing rate of STN neurons was significantly higher after 5-HT depletion (firing rate = 7.48 ± 0.50 Hz for the vehicle-treated control vs. 10.26 ± 0.60 Hz for the pCPA-treated group; *p* < 0.001, unpaired Student’s *t* test), whereas it remained unchanged after prolonged treatment with fluoxetine (firing rate = 7.33 ± 0.62 Hz for the vehicle-treated, and 7.70 ± 0.55 Hz for the fluoxetine-treated group; *p* > 0.05, unpaired Student’s *t* test). No differences were found in the coefficient of variation of the recorded STN neurons from the different groups (Fig. 1b). However, 5-HT depletion also modified the firing pattern of STN neurons, increasing the relative amount of STN neurons that exhibited bursting firing pattern comparing to its control group (53.65 % in pCPA-treated rats vs. 29.31 % in vehicle-treated control rats; *p* < 0.01, Fisher’s exact test; Fig. 1c). Finally, no differences in the firing pattern between vehicle and fluoxetine-treated groups were observed (Fig. 1c).

On the other hand, pCPA treatment reduced the concentrations of 5-HT in the STN (*F*_(2,14)_ = 27.51, *p* < 0.001, one-way ANOVA) and of the main 5-HT metabolite, 5-HIAA (*F*_(2,14)_ = 58.14, *p* < 0.001, one-way ANOVA; Fig. 1d). The levels of DA were not modified in any of the experimental groups (*F*_(2,14)_ = 0.09, *p* > 0.05, one-way ANOVA). Immunostaining experiments showed no difference in the density of SERT between vehicle-control and pCPA-treated rats in dorsal raphe nucleus target areas (prefrontal cortex and hippocampus), neither in the STN (Figure S3A, B).

Finally, the intravascular administration of the non-selective serotonergic receptor antagonist, methiothepin (25–1,000 μg/kg, i.v., *n* = 9) did not produce any significant modification in the firing rate (*F*_(6, 43)_ = 0.353, *p >* 0.05, one-way ANOVA), nor in the coefficient of variation (*F*_(6,43)_ = 0.693, *p >* 0.05 one-way ANOVA) of STN neurons in control rats (Table 1). However, methiothepin administration increased the number of neurons exhibiting a bursting firing pattern (*p* < 0.001, Fisher’s exact test; Table 1).

**Table 1:** Effects of systemic administration of cumulative doses of the non-specific 5-HT antagonist, methiothepin (*n* = 9 neurons from nine rats), the 5-HT_1A_ antagonist, WAY 100635 (*n* = 7 neurons from seven rats), and the 5-HT_2C_ antagonist, SB 242084 (*n* = 6 neurons from six rats) on STN neuron activity in control rats

Drug	Dose (μg/kg, i.v.)	Firing rate (%)	CV (%)	Firing pattern (%)		
				Tonic	Random	Bursting
Methiothepin	0	100	100	55.6	11.1	33.3
	25	110.1 ± 11.97	105.3 ± 10.18	22.2	0	77.8***
	50	120.2 ± 17.40	114.9 ± 13.50	33.3	11.1	55.6***
	100	118.2 ± 19.11	123.4 ± 16.39	55.6	11.1	33.3
	200	132.7 ± 17.91	117.4 ± 13.61	22.2	11.1	66.7***
	400	111.3 ± 7.75	146.8 ± 12.53	22.2	11.1	66.7***
	1,000	126.7 ± 22.89	135.2 ± 58.28	22.2	11.1	66.6***
WAY 100635	0	100	100	42.9	14.2	42.9
	6.25	87.6 ± 9.7	107.9 ± 9.9	14.2	14.2	71.6***
	12.5	90.0 ± 14.1	104.4 ± 11.7	14.3	0	85.7***
	25	99.1 ± 18.6	108.4 ± 17.4	0	0	100***
	50	104.3 ± 17.5	114.6 ± 16.4	14.3	0	85.7***
	75	106.8 ± 18.7	114.1 ± 17.7	14.3	0	85.7***
	100	114.9 ± 20.1	107.7 ± 16.7	42.9	14.2	42.9
SB 242084	0	100	100	83.3	0	16.7
	12.5	98.9 ± 1.0	100.6 ± 3.3	66.7	0	33.3
	25	102.3 ± 3.5	100.0 ± 3.3	83.3	0	16.7
	50	101.5 ± 3.4	105 ± 6.3	66.7	0	33.3
	100	108.2 ± 9.2	106.2 ± 5.4	66.7	0	33.3
	150	106.0 ± 7.7	102.5 ± 7.2	83.3	0	16.7
	200	106.9 ± 9.1	103.0 ± 9.8	66.7	0	33.3

Data from the firing rate and the coefficient of variation (CV) are expressed as means ± SEM of the percentage of the basal condition. The firing pattern is presented as percentage of neurons which represent the standard discharge patterns of STN neurons

***p < 0.001 vs. basal condition (Fisher’s exact test)

### Effect of 5-HT_1A_ serotonergic drugs on STN neuron activity in control and pCPA-treated rats

**Fig. 2:**
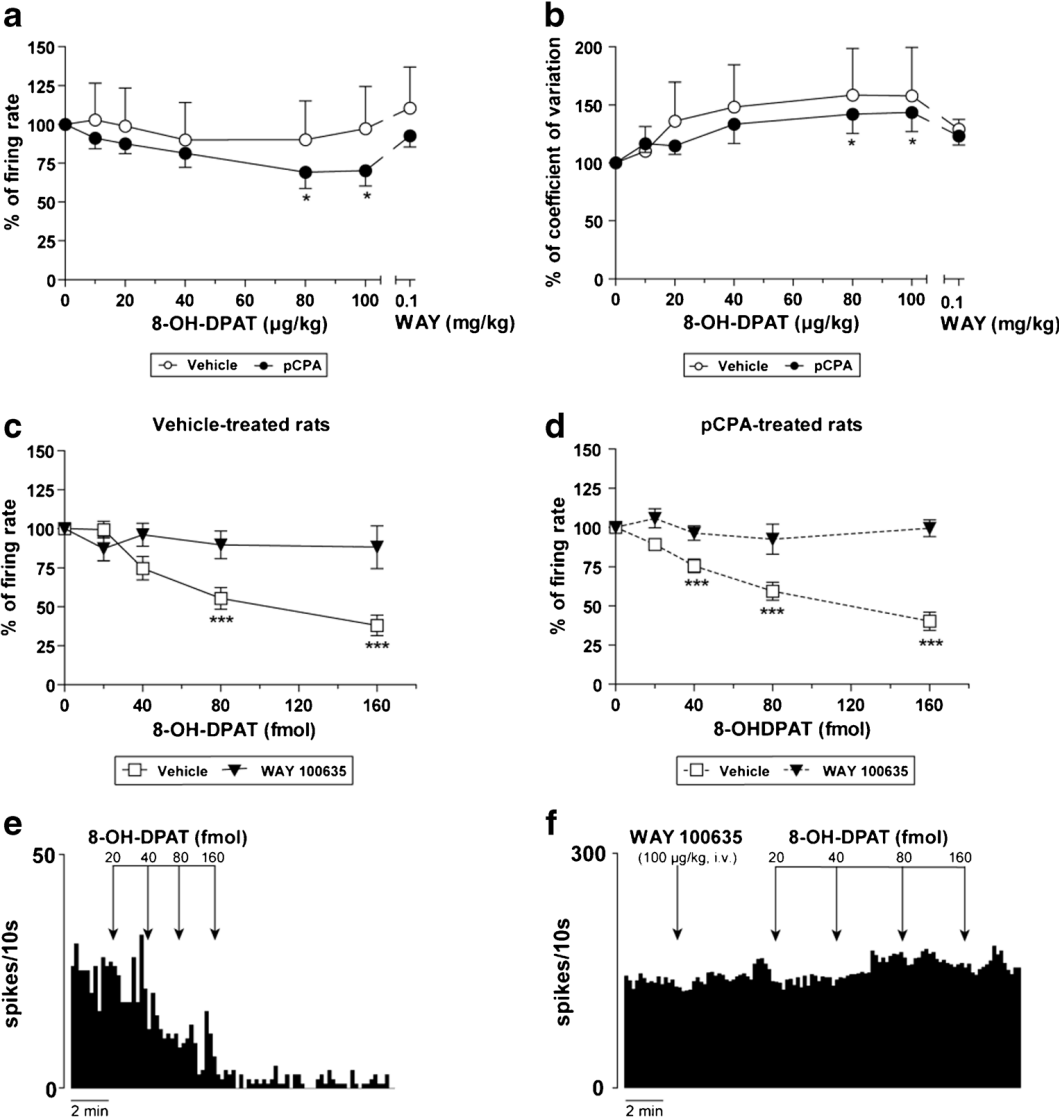
Effect of 8-OH-DPAT on STN neuron activity. 8-OH-DPAT (10–100 μg/kg, i.v.) induced an inhibition in the firing rate (**a**) and an increase in the coefficient of variation (**b**) in pCPA-treated (300 mg/kg/day, i.p. 3 days) rats (*black circles*; *n* = 8 neurons from eight rats). This effect was reverted by administration of the 5-HT_1A_ antagonist, WAY 100635 (0.1 mg/kg, i.v.). In vehicle-control rats (*white circles*; *n* = 8 neurons from eight rats), firing rate, and coefficient of variation remained unaltered (**a** and **b**). **c** When 8-OH-DPAT (20–160 fmol) was locally applied into the STN (*white square*; *n* = 12 neurons from seven vehicle-control rats) an inhibitory effect was induced. The systemic administration of WAY 100635 (0.1 mg/kg, i.v.) prior to the local application of 8-OH-DPAT blocked the inhibitory effect of the 5-HT_1A_ receptor agonist (*black triangle*; *n* = 11 neurons from 11 vehicle-control rats). **d** 8-OH-DPAT (20–160 fmol) locally applied into the STN of pCPA-treated animals (*white square*; *n* = 26 neurons from five pCPA-treated rats) caused an inhibitory effect. The systemic administration of WAY 100635 (0.1 mg/kg, i.v.) prior to the local application of 8-OH-DPAT blocked the inhibitory effect of the 5-HT_1A_ receptor agonist (*black triangle*; *n* = 5 neurons from five pCPA-treated rats). **e** Representative firing rate histogram illustrating the inhibitory effect of local administration of 8-OH-DPAT on STN neuronal activity. **f** Representative firing rate histogram illustrating WAY 100635 induced blockage of the inhibitory effect of local administration of 8-OH-DPAT on STN neuron activity. *p < 0.05 and ***p < 0.001 vs. basal firing rate (Bonferroni’s post hoc test)

Systemic administration of cumulative doses of the selective 5-HT_1A_ agonist, 8-OH-DPAT (10–100 μg/kg, i.v., *n* = 8) did not change the firing rate of STN neurons or the coefficient of variation in vehicle-control group rats (Fig. 2a, b). However, in pCPA-treated rats, 8-OH-DPAT reduced, in a dose-dependent manner, the firing rate by 30 % (*F*_(5,35)_ = 5.961, *p* < 0.001, RM one-way ANOVA) and increased the coefficient of variation by 44 % (*F*_(5,35)_ = 4.255, *p* < 0.01, RM one-way ANOVA; Fig. 2a, b). This effect was reversed by the 5-HT_1A_ antagonist, WAY 100635 (100 μg/kg, i.v., *n* = 8; Fig. 2a, b). No effect on the firing pattern of STN neurons was observed after 8-OH-DPAT administration in either vehicle- or pCPA-treated groups (*p* > 0.05, Fisher’s exact test).

Next, 8-OH-DPAT (20–160 fmol) applied directly into the STN caused a marked inhibition of STN neuron activity in vehicle-control animals [maximal reduction of the firing rate was 62 % of the basal value (*F*_(4,42)_ = 17.85, *p* < 0.001, one-way ANOVA; Fig. 2c, e)], and in pCPA-treated animals [maximal reduction of the firing rate was 60 % of the basal value (*F*_(4,119)_ = 30.46, *p* < 0.001, one-way ANOVA; Fig. 2d, e)]. This inhibitory effect of 8-OH-DPAT (20–160 fmol) was blocked by the previous systemic administration of WAY 100635 (100 μg/kg, i.v), in both vehicle-control (*n* = 10) and pCPA-treated (*n* = 5) rats (Fig. 2f).

Finally, WAY 100635 did not modify the firing rate or the coefficient of variation of STN neurons, neither when WAY 100635 was administrated systemically (6.25–100 μg/kg, i.v., *n* = 7; Table 1) nor when it was locally applied into the STN (20–320 fmol, *n* = 51 neurons from eight rats) (Figure S1A). However, the intravascular administration of cumulative doses of WAY 100635 enhanced the amount of STN neurons that exhibited a bursting firing pattern (*p <* 0.001, Fisher’s exact test; Table 1). After pCPA treatment WAY 100635 did not elicit any effect on STN neuron activity (data not shown).

### Effect of 5-HT_2C_ serotonergic drugs on STN neuron activity in control and pCPA-treated rats

**Fig. 3:**
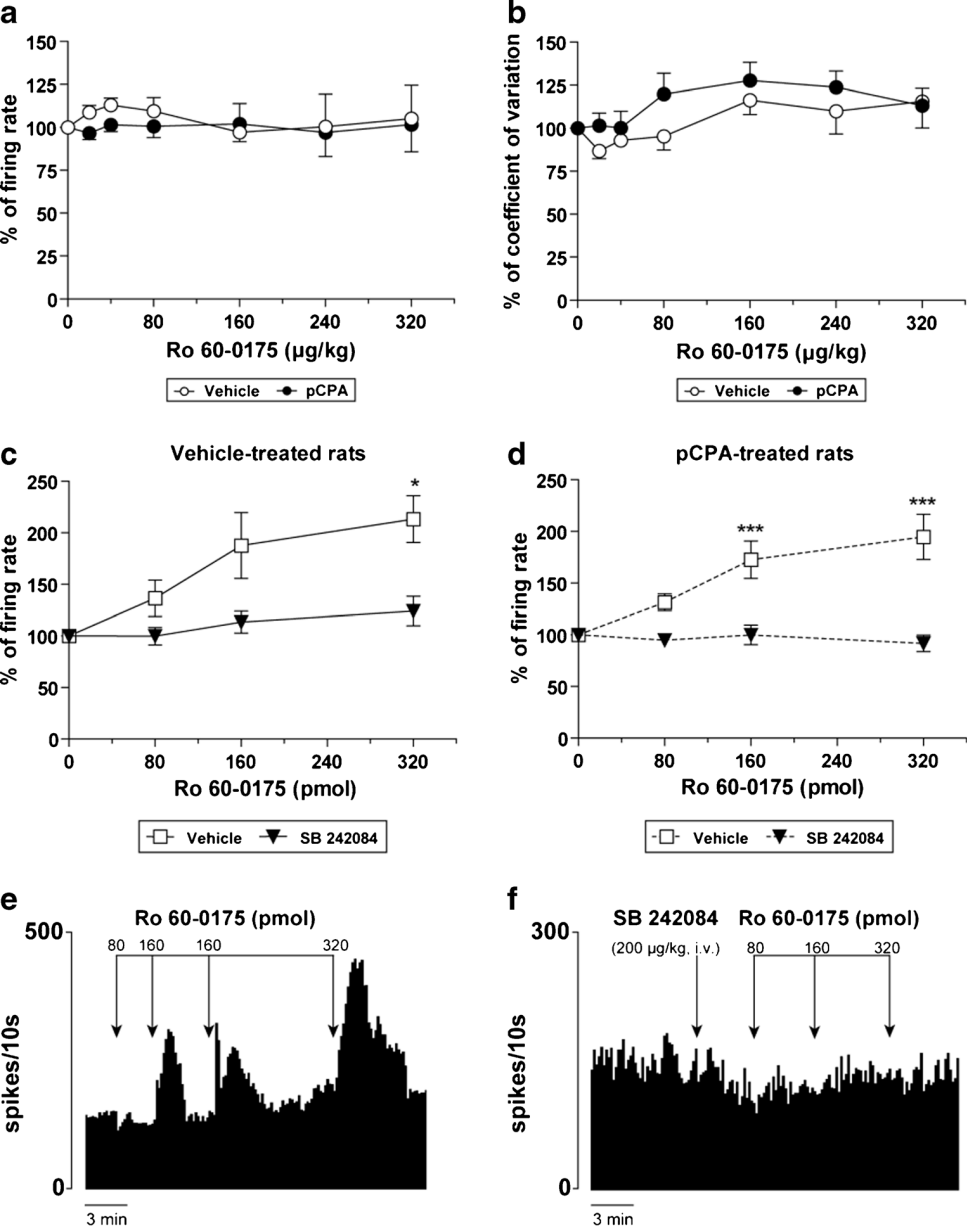
Effect of Ro 60-0175 on STN neuron activity. Intravenous administration of Ro 60-0175 (20–320 μg/kg) did not induce any effect on the firing rate (**a**) or on the coefficient of variation (**b**) either in pCPA- (300 mg/kg/day, i.p. 3 days; *black circle*; *n* = 8 neurons from eight rats) or vehicle-treated rats (*white circle*; *n* = 6 neurons from six rats). **c** When Ro 60-0175 (80–320 pmol) was locally applied into the STN (*white square*; *n* = 11 neurons from five vehicle-control rats) a stimulatory effect was induced. The systemic administration of SB 242084 (0.2 mg/kg, i.v.) prior to the local application of Ro 60-0175 blocked the stimulatory effect of the 5-HT_2C_ receptor agonist (*black triangle*; *n* = 14 neurons from 14 vehicle-control rats). **d** Ro 60-0175 locally applied into the STN of pCPA-treated animals (*white square*; *n* = 36 neurons from seven pCPA-treated rats) caused a stimulatory effect. The systemic administration of SB 242084 (0.2 mg/kg, i.v.) prior to the local application of Ro 60-0175 blocked the stimulatory effect of the 5-HT_2C_ receptor agonist (*black triangle*; *n* = 7 neurons from seven pCPA-treated rats). **e** Representative firing rate histogram illustrating the stimulatory effect of local administration of Ro 60-0175 on STN neuronal activity. **f** Representative firing rate histogram illustrating SB 242084 induced blockage of the stimulatory effect of local administration of Ro 60-0175 on STN neuron activity. *p < 0.05 and ***p < 0.001 vs. basal firing rate (Bonferroni’s post hoc test)

The administration of cumulative doses of the 5-HT_2C_ agonist, Ro 60-0175 (20–320 μg/kg, i.v.), did not affect the firing parameters of STN neurons in vehicle- (*n* = 6) or pCPA-treated rats (*n* = 8; Fig. 3a, b). However, as shown in Fig. 3c and d, local application of Ro 60-0175 (80–320 pmol) into the STN markedly stimulated the recorded neurons from vehicle-control animals [maximal increment of the firing rate was 113 % of the basal value (*F*_(3,33)_ = 4.49, *p* < 0.01, one-way ANOVA)] and from pCPA-treated animals [maximal increment of the firing rate was 95 % of the basal value (*F*_(3,116)_ = 11.23, *p* < 0.001, one-way ANOVA)]. Additionally, the systemic administration of the 5-HT_2C_ antagonist, SB 242084 (200 μg/kg, i.v.) prior to the local application of Ro 60-0175 into the STN blocked the stimulatory effect of the 5-HT_2C_ receptor agonist, both in vehicle-control (*n* = 11) and in pCPA-treated (*n* = 7) rats (Fig. 3f).

The systemic administration of SB 242084 (12.5–200 μg/kg, i.v.; *n* = 6), did not modify any firing parameter of STN neurons in control rats (Table 1). Moreover, the local application of SB 242084 (200–3,200 fmol, *n* = 34 neurons from five rats) into the STN neither changed the firing rate in control rats (Figure S1B). After pCPA treatment SB 242084 did not elicit any effect on STN neuron activity (data not shown).

### Effect of fluoxetine treatment on STN neuron response to fluoxetine, 5HT_1A_, and 5HT_2C_ agonists and motor behavior

**Fig. 4:**
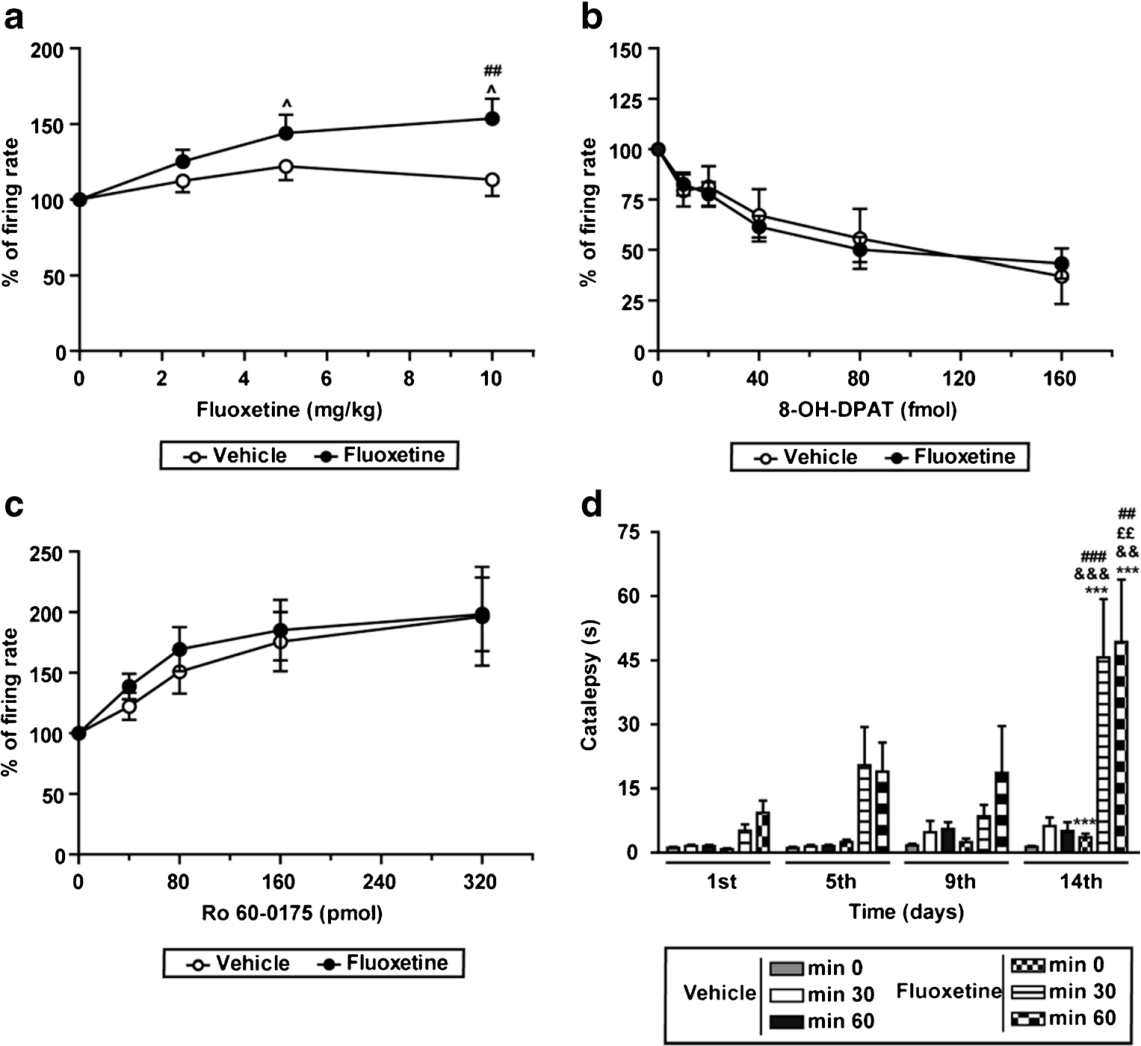
Effect of fluoxetine on STN neuron activity and motor behavior in vehicle- and chronic fluoxetine-treated (10 mg/kg/day, i.p. 14 days) rats. **a** Cumulative doses of fluoxetine (2.5–10 mg/kg, i.v.) induced an increase in the firing rate in fluoxetine-treated rats (*black circle*; *n* = 8 neurons from eight fluoxetine-treated rats), but not in the vehicle-treated rats (*white circle*; *n* = 14 neurons from 14 vehicle-treated control rats). The fluoxetine treatment did not modify the inhibitory (**b**) and the excitatory (**c**) effects induced by the local application of 8-OH-DPAT (20–160 fmol) or Ro 60-0175 (40–320 pmol) into the STN, respectively. **d** Fluoxetine chronic administration induced catalepsy on day 14 after the beginning of the treatment. Note that no catalepsy was observed before fluoxetine administration or at any time in the vehicle-treated group. Eight animals were included in each group. ^*p* < 0.05 vs. basal firing rate; ^##^ *p <* 0.01 and ^###^ *p* < 0.001 vs. respective vehicle-treated group; ***p < 0.001 vs. respective first day; ^££^ *p <* 0.01 vs. respective fifth day; &&*p <* 0.01 and &&&*p <* 0.001 vs. respective ninth day (Bonferroni’s post hoc test)

In vehicle-treated control rats, the acute administration of fluoxetine (2.5–10 mg/kg, i.v.; *n* = 14) did not induce any change in STN activity (Fig. 4a). However, after 14 days of treatment with fluoxetine (10 mg/kg every 24 h, i.p.), a challenge of fluoxetine (*n* = 8) produced a significant increase of 53 % of the firing rate (*F*_(3,21)_ = 9.141, *p* < 0.001, RM one-way ANOVA; Fig. 4a), while the coefficient of variation (54.64 ± 6.68 % basal condition vs. 44.24 ± 2.24 % after 10 mg/kg dose) and the firing pattern (44.0 % of bursting neurons in basal condition vs. 35.5 % of bursting neurons after 10 mg/kg dose) remained unaltered. The fluoxetine treatment did not change the inhibitory effect induced on STN neurons by the local application into the STN of the 5-HT_1A_ receptor agonist, 8-OH-DPAT (20–160 fmol, *n* = 29 neurons from nine rats) (*F*_(1,208)_ = 0.71, *p >* 0.05, two-way ANOVA; Fig. 4b), nor the excitatory effect induced by the 5-HT_2C_ receptor agonist, Ro 60-0175 (40–320 pmol, *n* = 35 neurons from nine rats; *F*_(1,202)_ = 0.52, *p >* 0.05, two-way ANOVA; Fig. 4c).

Finally, the bar test and the rotarod test were performed to analyze if the fluoxetine treatment induced motor impairments. Fluoxetine treatment did not produce any modification in the rotarod test (Figure S2A). However, on the course of the chronic treatment, fluoxetine-treated animals showed marked catalepsy in comparison to the vehicle-injected group both 30 (*F*_(1,42)_ = 10.84, *p <* 0.01, RM two-way ANOVA) and 60 min after the administration (*F*_(1,42)_ = 7.62, *p <* 0.05, RM two-way ANOVA). On day 14, rats in the fluoxetine-treated group remained significantly longer time on the bar (for statistical details see Fig. 4d). In agreement with other authors (Gutierrez et al. 2002), fluoxetine chronic treatment had an anorexigenic effect and rats belonging to that group showed reduction of body weight in comparison to the vehicle-treated control rats (Figure S2B).

## Discussion

The results of the present study show that 5-HT depletion as well as systemic administration of either a non-selective serotonergic receptor antagonist or a 5-HT_1A_ receptor antagonist changed the firing pattern of STN neurons. Moreover, disruption of the endogenous serotonergic tonic control results in an increment of STN neuron activity. Local activation of 5-HT_1A_ and 5-HT_2C_ receptors leads to opposite effects, i.e., inhibition and stimulation, on STN neuron activity. Finally, the 5-HT_1A_-mediated effect depends on the tissue 5-HT content and prolonged 5-HT reuptake inhibition enhances STN neuronal response to fluoxetine but induces cataleptic behavior in rats.

The main serotonergic modulation of STN activity arises from the dorsal raphe nucleus, which imposes a tonic inhibition of basal STN activity (Di Matteo et al. 2008). In agreement with our results, lesioning the dorsal raphe nucleus with 5,7-dihydroxytryptamine results in an increase in the firing rate and in the number of bursting neurons in the STN in rats (Liu et al. 2007). In addition to the direct serotonergic control from the dorsal raphe nucleus, indirect serotonergic mechanisms may also control STN activity, since several nuclei projecting to the STN are regulated by 5-HT and express serotonergic receptors (Huot et al. 2011; Liu et al. 2007).

In the present study, we observed that after 5-HT depletion, but not in control conditions, systemic administration of the selective 5-HT_1A_ agonist, 8-OH-DPAT, inhibited STN neuron activity. This finding may be attributed to the availability of 5-HT, since no changes in the sensitivity of 5-HT_1A_ receptors to 8-OH-DPAT have been reported after pCPA treatment (Eide et al. 1988). Moreover, when 8-OH-DPAT was applied directly into the STN, it produced a larger inhibition than that observed by systemic administration. This inhibitory effect was mediated by 5-HT_1A_ receptors as it was reversed or blocked by systemic administration of the selective 5-HT_1A_ receptor antagonist, WAY 100635. Altogether, these results indicate that the effect of 8-OH-DPAT on the STN involves direct and indirect mechanisms: (1) a direct effect mediated by the activation of postsynaptic 5-HT_1A_ receptors on STN neurons that results in the inhibition of STN neurons, as it is suggested by in vitro experiments (Shen et al. 2007; Stanford et al. 2005) and (2) an indirect effect by activating 5-HT_1A_ receptors on the dorsal raphe nucleus (that reduces the release of 5-HT) and areas that project to the STN. The activation of serotonergic receptors on those nuclei may hide the direct inhibitory effect mediated by 5-HT_1A_ receptors on STN neurons.

Systemic administration of the 5-HT_2C_ agonist Ro 60-0175 did not modify STN activity in control conditions or after 5-HT depletion, while local application stimulated STN neurons. This stimulation was blocked by systemic administration of the selective 5-HT_2C_ receptor antagonist SB 242084. Although these results agree with in vitro studies, where 5-HT_2C_ receptors has been proposed to mediate stimulation of STN neurons (Shen et al. 2007; Stanford et al. 2005), they differ from those published by Zhang et al. (2009), where an increase in the firing rate of STN neurons was reported after systemic administration of a non-selective 5-HT_2C_ agonist. The discrepancies with the latter publication may reflect the different selectivity of the applied drugs and highlight the importance of using specific drugs. Our results suggest that 5-HT_2C_ receptors located in the STN, and in other brain areas, are implicated in the modulation of neuronal firing rate, as it has been described in other BG nuclei (Beyeler et al. 2010; Invernizzi et al. 2007). As also mentioned for 5-HT_1A_ receptors, the activation of 5-HT_2C_ located outside the STN may blunt the direct effect of the 5-HT_2C_ receptor agonist when it is systemically applied.

We observed that chronic fluoxetine treatment did not change the spontaneous activity of STN neurons or the basal 5-HT levels in this nucleus, but it enhanced the sensitivity to fluoxetine. In line with this, using microdialysis techniques, Dawson et al. (2000, 2002) have shown that chronic fluoxetine treatment increases extracellular 5-HT in response to a fluoxetine challenge, despite not altering basal 5-HT levels in the frontal cortex. We also found that chronic fluoxetine treatment did not modify the effect of local application of 5-HT_1A_ and 5-HT_2C_ receptor agonists in the STN. This suggests that chronic fluoxetine treatment does not change the sensitivity of postsynaptic 5-HT_1A_ and 5-HT_2C_ receptors in STN neurons, as it has been proposed for other brain areas (Le Poul et al. 2000). In contrast, other authors have described a desensitization of somatodendritic 5-HT_1A_ receptors after fluoxetine treatment (Blier and de Montigny 1994; Dawson et al. 2000, 2002; Le Poul et al. 2000; Rainer et al. 2012). Freo et al. (2010) have pointed that chronic fluoxetine administration reduces and increases the response mediated by 5-HT_1A_ and 5-HT_2A/2C_ receptors, respectively. These effects are not homogeneous through the brain, being observed in some areas but not in others. As mentioned before, the enhancement of STN neuron activity induced by systemic fluoxetine administration in fluoxetine-treated rats could be a consequence of effects in other brain areas. In fact, an acute challenge of fluoxetine in chronically treated animals gives rise to increased extracellular 5-HT levels in the frontal cortex (Dawson et al. 2000, 2002) and reduced DA levels in the dorsal striatum (Morelli et al. 2011). Given that these areas influence STN activity, such changes in neurotransmitter concentrations may be related to the electrophysiological changes observed in the STN and in motor behavior.

Using behavioral approaches, we found that chronic fluoxetine treatment did not impair motor coordination and balance in rats, although it induced catalepsy. Other authors have reported that acute fluoxetine administration potentiates haloperidol-induced catalepsy (Tatara et al. 2012) and that the STN is implicated in cataleptic behavior (Zadow and Schmidt 1994). According to the widely accepted BG functional model (Alexander et al. 1986), increased STN neuron activity results in an enhancement of BG output nuclei signal leading to movement inhibition and therefore, movement disorders (Hamani et al. 2004). Hence, we speculate that the increment in STN activity is involved in the extrapyramidal side effects induced by fluoxetine.

In conclusion, this study shows that 5-HT modifies STN neuron activity through direct and indirect mechanisms. Our results contribute to clarify the role of serotonergic neurotransmission in STN neuron activity and understanding the mechanisms underlying the extrapyramidal side effects induced by SSRIs.

## Electronic supplementary material

Below is the link to the electronic supplementary material.

Supplementary Figure S1 (DOC 241 kb)

Supplementary Figure S2 (DOC 271 kb)

Supplementary Figure S3 (DOC 2334 kb)

## Abbreviations

BG: Basal ganglia
STN: Subthalamic nucleus
pCPA: 4-chloro-DL-phenylalanine methyl ester hydrochloride
5-HT: Serotonin
5-HIAA: 5-hydroxy-indole-acetic acid
SSRI: Serotonin selective reuptake inhibitor
DA: Dopamine

## References

[CR1] Alexander GE, DeLong MR, Strick PL (1986). Parallel organization of functionally segregated circuits linking basal ganglia and cortex. Annu Rev Neurosci.

[CR2] Belforte JE, Pazo JH (2004). Turning behaviour induced by stimulation of the 5-HT receptors in the subthalamic nucleus. Eur J Neurosci.

[CR3] Benarroch EE (2009). Serotonergic modulation of basal ganglia circuits: complexity and therapeutic opportunities. Neurology.

[CR4] Beyeler A, Kadiri N, Navailles S, Boujema MB, Gonon F, Moine CL, Gross C, De Deurwaerdere P (2010). Stimulation of serotonin2C receptors elicits abnormal oral movements by acting on pathways other than the sensorimotor one in the rat basal ganglia. Neuroscience.

[CR5] Bilen S, Saka M, Ak F, Oztekin N (2008). Persistent dystonia induced by fluoxetine. Intern Med J.

[CR6] Blier P, de Montigny C (1994). Current advances and trends in the treatment of depression. Trends Pharmacol Sci.

[CR7] Cordero MI, Rodriguez JJ, Davies HA, Peddie CJ, Sandi C, Stewart MG (2005). Chronic restraint stress down-regulates amygdaloid expression of polysialylated neural cell adhesion molecule. Neuroscience.

[CR8] Crossman AR (2000). Functional anatomy of movement disorders. J Anat.

[CR9] Dawson LA, Nguyen HQ, Smith DI, Schechter LE (2000). Effects of chronic fluoxetine treatment in the presence and absence of (+/−)pindolol: a microdialysis study. Br J Pharmacol.

[CR10] Dawson LA, Nguyen HQ, Smith DL, Schechter LE (2002). Effect of chronic fluoxetine and WAY-100635 treatment on serotonergic neurotransmission in the frontal cortex. J Psychopharmacol.

[CR11] De Deurwaerdere P, Chesselet MF (2000). Nigrostriatal lesions alter oral dyskinesia and c-Fos expression induced by the serotonin agonist 1-(m-chlorophenyl)piperazine in adult rats. J Neurosci.

[CR12] Dekundy A, Pietraszek M, Schaefer D, Cenci MA, Danysz W (2006). Effects of group I metabotropic glutamate receptors blockade in experimental models of Parkinson's disease. Brain Res Bull.

[CR13] Di Matteo V, Pierucci M, Esposito E, Crescimanno G, Benigno A, Di Giovanni G (2008). Serotonin modulation of the basal ganglia circuitry: therapeutic implication for Parkinson's disease and other motor disorders. Prog Brain Res.

[CR14] Eberle-Wang K, Lucki I, Chesselet MF (1996). A role for the subthalamic nucleus in 5-HT2C-induced oral dyskinesia. Neuroscience.

[CR15] Eberle-Wang K, Mikeladze Z, Uryu K, Chesselet MF (1997). Pattern of expression of the serotonin2C receptor messenger RNA in the basal ganglia of adult rats. J Comp Neurol.

[CR16] Eide PK, Hole K, Berge OG, Broch OJ (1988). 5-HT depletion with 5,7-DHT, PCA and PCPA in mice: differential effects on the sensitivity to 5-MeODMT, 8-OH-DPAT and 5-HTP as measured by two nociceptive tests. Brain Res.

[CR17] Flores G, Rosales MG, Hernandez S, Sierra A, Aceves J (1995). 5-Hydroxytryptamine increases spontaneous activity of subthalamic neurons in the rat. Neurosci Lett.

[CR18] Freo U, Merico A, Ermani M, Ori C (2010). Chronic treatment with fluoxetine decreases cerebral metabolic responses to the 5-HT1A agonist 8-hydroxy-2(di-*N*-propylamino)tetralin and increases those to the 5-HT2A/2C agonist 1-(2,5-dimethoxy-4-iodophenyl)-2-aminopropane and to the dopaminergic agonist apomorphine. Brain Res.

[CR19] Grossman CJ, Kilpatrick GJ, Bunce KT (1993). Development of a radioligand binding assay for 5-HT4 receptors in guinea-pig and rat brain. Br J Pharmacol.

[CR20] Guirado R, Varea E, Castillo-Gomez E, Gomez-Climent MA, Rovira-Esteban L, Blasco-Ibanez JM, Crespo C, Martinez-Guijarro FJ, Nacher J (2009). Effects of chronic fluoxetine treatment on the rat somatosensory cortex: activation and induction of neuronal structural plasticity. Neurosci Lett.

[CR21] Gutierrez A, Saracibar G, Casis L, Echevarria E, Rodriguez VM, Macarulla MT, Abecia LC, Portillo MP (2002). Effects of fluoxetine administration on neuropeptide y and orexins in obese zucker rat hypothalamus. Obes Res.

[CR22] Hamani C, Saint-Cyr JA, Fraser J, Kaplitt M, Lozano AM (2004). The subthalamic nucleus in the context of movement disorders. Brain.

[CR23] Hannon J, Hoyer D (2008). Molecular biology of 5-HT receptors. Behav Brain Res.

[CR24] Hollerman JR, Grace AA (1992). Subthalamic nucleus cell firing in the 6-OHDA-treated rat: basal activity and response to haloperidol. Brain Res.

[CR25] Hoyer D, Hannon JP, Martin GR (2002). Molecular, pharmacological and functional diversity of 5-HT receptors. Pharmacol Biochem Behav.

[CR26] Huot P, Fox SH, Brotchie JM (2011). The serotonergic system in Parkinson's disease. Prog Neurobiol.

[CR27] Invernizzi RW, Pierucci M, Calcagno E, Di Giovanni G, Di Matteo V, Benigno A, Esposito E (2007). Selective activation of 5-HT(2C) receptors stimulates GABA-ergic function in the rat substantia nigra pars reticulata: a combined in vivo electrophysiological and neurochemical study. Neuroscience.

[CR28] Kaneoke Y, Vitek JL (1996). Burst and oscillation as disparate neuronal properties. J Neurosci Methods.

[CR29] Lavoie B, Parent A (1990). Immunohistochemical study of the serotoninergic innervation of the basal ganglia in the squirrel monkey. J Comp Neurol.

[CR30] Le Poul E, Boni C, Hanoun N, Laporte AM, Laaris N, Chauveau J, Hamon M, Lanfumey L (2000). Differential adaptation of brain 5-HT1A and 5-HT1B receptors and 5-HT transporter in rats treated chronically with fluoxetine. Neuropharmacology.

[CR31] Lee LJ (2012). Neonatal fluoxetine exposure alters motor performances of adolescent rats. Dev Neurobiol.

[CR32] Leo RJ (1996). Movement disorders associated with the serotonin selective reuptake inhibitors. J Clin Psychiatry.

[CR33] Liu J, Chu YX, Zhang QJ, Wang S, Feng J, Li Q (2007). 5,7-dihydroxytryptamine lesion of the dorsal raphe nucleus alters neuronal activity of the subthalamic nucleus in normal and 6-hydroxydopamine-lesioned rats. Brain Res.

[CR34] Maroteaux L, Saudou F, Amlaiky N, Boschert U, Plassat JL, Hen R (1992). Mouse 5HT1B serotonin receptor: cloning, functional expression, and localization in motor control centers. Proc Natl Acad Sci U S A.

[CR35] Martinez-Price DL, Geyer MA (2002). Subthalamic 5-HT(1A) and 5-HT(1B) receptor modulation of RU 24969-induced behavioral profile in rats. Pharmacol Biochem Behav.

[CR36] Miguelez C, Aristieta A, Cenci MA, Ugedo L (2011). The locus coeruleus is directly implicated in L-DOPA-induced dyskinesia in parkinsonian rats: an electrophysiological and behavioural study. PLoS One.

[CR37] Morelli E, Moore H, Rebello TJ, Gray N, Steele K, Esposito E, Gingrich JA, Ansorge MS (2011). Chronic 5-HT transporter blockade reduces DA signaling to elicit basal ganglia dysfunction. J Neurosci.

[CR38] Morera-Herreras T, Ruiz-Ortega JA, Taupignon A, Baufreton J, Manuel I, Rodriguez-Puertas R, Ugedo L (2010). Regulation of subthalamic neuron activity by endocannabinoids. Synapse.

[CR39] Obeso JA, Rodriguez-Oroz MC, Rodriguez M, Lanciego JL, Artieda J, Gonzalo N, Olanow CW (2000). Pathophysiology of the basal ganglia in Parkinson's disease. Trends Neurosci.

[CR40] Palkovits M, Brownstein M, Saavedra JM (1974). Serotonin content of the brain stem nuclei in the rat. Brain Res.

[CR41] Paxinos G, Watson C (1997) The rat brain in stereotaxic coordinates (3rd edn). Academic, New York

[CR42] Pazos A, Palacios JM (1985). Quantitative autoradiographic mapping of serotonin receptors in the rat brain. I. Serotonin-1 receptors. Brain Res.

[CR43] Pazos A, Cortes R, Palacios JM (1985). Quantitative autoradiographic mapping of serotonin receptors in the rat brain. II. Serotonin-2 receptors. Brain Res.

[CR44] Pompeiano M, Palacios JM, Mengod G (1992). Distribution and cellular localization of mRNA coding for 5-HT1A receptor in the rat brain: correlation with receptor binding. J Neurosci.

[CR45] Rainer Q, Nguyen HT, Quesseveur G, Gardier AM, David DJ, Guiard BP (2012). Functional status of somatodendritic serotonin 1A autoreceptor after long-term treatment with fluoxetine in a mouse model of anxiety/depression based on repeated corticosterone administration. Mol Pharmacol.

[CR46] Rodriguez JJ, Jones VC, Tabuchi M, Allan SM, Knight EM, LaFerla FM, Oddo S, Verkhratsky A (2008). Impaired adult neurogenesis in the dentate gyrus of a triple transgenic mouse model of Alzheimer's disease. PLoS One.

[CR47] Rodriguez JJ, Jones VC, Verkhratsky A (2009). Impaired cell proliferation in the subventricular zone in an Alzheimer's disease model. Neuroreport.

[CR48] Ruiz-Ortega JA, Ugedo L (1997). The stimulatory effect of clonidine on locus coeruleus neurons of rats with inactivated alpha 2-adrenoceptors: involvement of imidazoline receptors located in the nucleus paragigantocellularis. Naunyn Schmiedebergs Arch Pharmacol.

[CR49] Saavedra JM (1977). Distribution of serotonin and synthesizing enzymes in discrete areas of the brain. Fed Proc.

[CR50] Sari Y, Miquel MC, Brisorgueil MJ, Ruiz G, Doucet E, Hamon M, Verge D (1999). Cellular and subcellular localization of 5-hydroxytryptamine1B receptors in the rat central nervous system: immunocytochemical, autoradiographic and lesion studies. Neuroscience.

[CR51] Shen KZ, Kozell LB, Johnson SW (2007). Multiple conductances are modulated by 5-HT receptor subtypes in rat subthalamic nucleus neurons. Neuroscience.

[CR52] Stanford IM, Kantaria MA, Chahal HS, Loucif KC, Wilson CL (2005). 5-Hydroxytryptamine induced excitation and inhibition in the subthalamic nucleus: action at 5-HT(2C), 5-HT(4) and 5-HT(1A) receptors. Neuropharmacology.

[CR53] Tatara A, Shimizu S, Shin N, Sato M, Sugiuchi T, Imaki J, Ohno Y (2012). Modulation of antipsychotic-induced extrapyramidal side effects by medications for mood disorders. Prog Neuropsychopharmacol Biol Psychiatry.

[CR54] Ugedo L, Grenhoff J, Svensson TH (1989). Ritanserin, a 5-HT2 receptor antagonist, activates midbrain dopamine neurons by blocking serotonergic inhibition. Psychopharmacology (Berl).

[CR55] Waeber C, Sebben M, Bock J, Dumuis A (1996). Regional distribution and ontogeny of 5-HT4 binding sites in rat brain. Behav Brain Res.

[CR56] Xiang Z, Wang L, Kitai ST (2005). Modulation of spontaneous firing in rat subthalamic neurons by 5-HT receptor subtypes. J Neurophysiol.

[CR57] Zadow B, Schmidt WJ (1994). Lesions of the entopeduncular nucleus and the subthalamic nucleus reduce dopamine receptor antagonist-induced catalepsy in the rat. Behav Brain Res.

[CR58] Zhang QJ, Liu X, Liu J, Wang S, Ali U, Wu ZH, Wang T (2009). Subthalamic neurons show increased firing to 5-HT2C receptor activation in 6-hydroxydopamine-lesioned rats. Brain Res.

