# Supplementary figures and images for: Modulation of the subthalamic nucleus activity by serotonergic agents and fluoxetine administration
Source: Psychopharmacology (Berl). 2013 Nov 24;231(9):1913–24. doi: 10.1007/s00213-013-3333-0 (PMC3984421; doi:10.1007/s00213-013-3333-0)

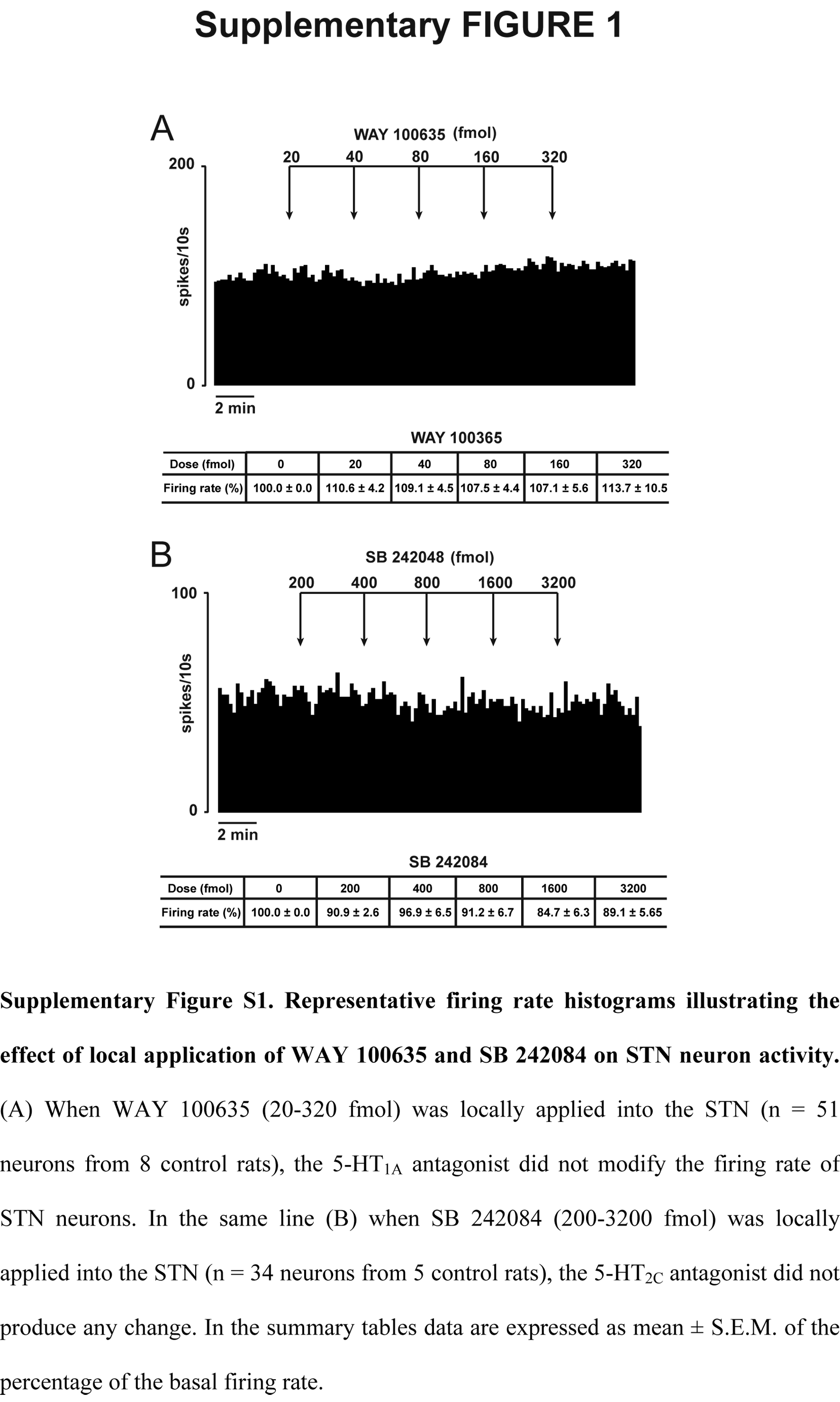

Supplement: Supplementary file 1 — (DOC 241 kb) [file 213_2013_3333_MOESM1_ESM.doc]

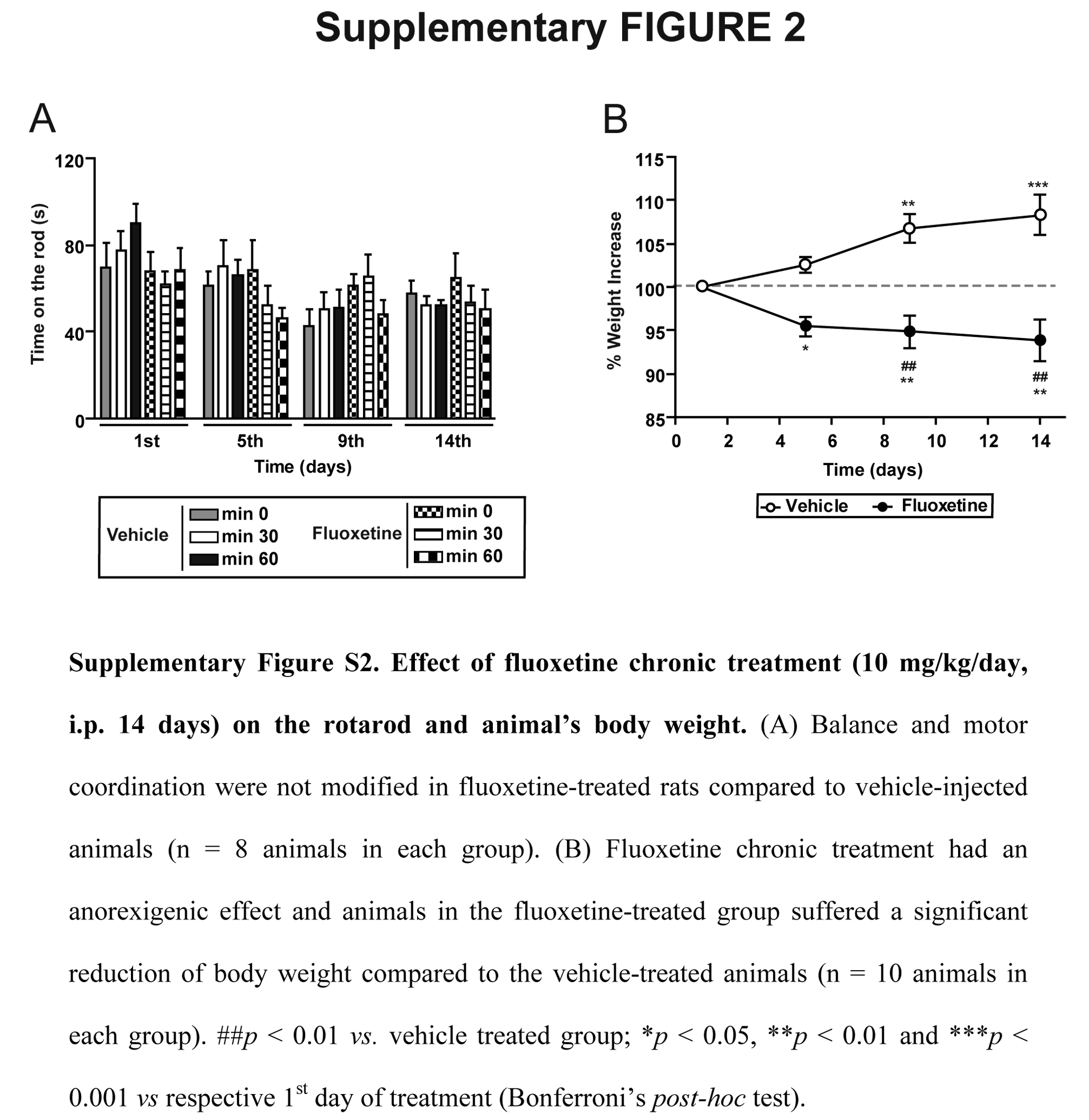

Supplement: Supplementary file 2 — (DOC 271 kb) [file 213_2013_3333_MOESM2_ESM.doc]

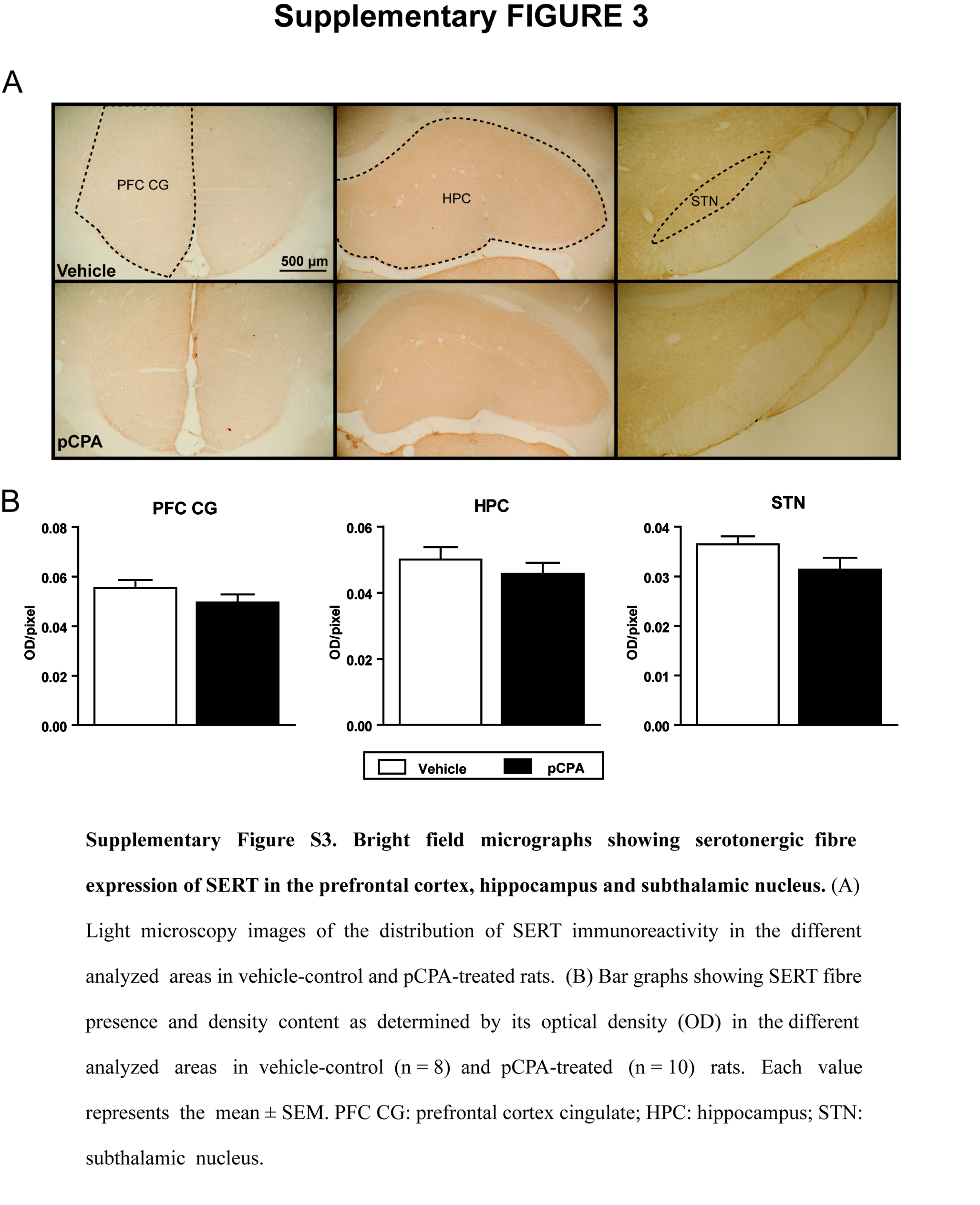

Supplement: Supplementary file 3 — (DOC 2334 kb) [file 213_2013_3333_MOESM3_ESM.doc]
